# Myocardial Infarction With Non-obstructive Coronary Arteries (MINOCA) in a High-Risk Young Man: A Case Report

**DOI:** 10.7759/cureus.85644

**Published:** 2025-06-09

**Authors:** Oumama Soussi, Houda Bachri, Nesma Bendagha, Aida Soufiani, Rokya Fellat

**Affiliations:** 1 Cardiology, Ibn Sina Hospital, Ibn Sina University Hospital Center, Mohammed V University, Rabat, MAR

**Keywords:** acute coronary syndrome (acs), cardiovascular risk factors (cvrf), myocardial infarction with non-obstructive coronary arteries (minocas), non-obstructive coronary disease, young adults

## Abstract

Acute coronary syndrome (ACS) in young adults with non-obstructive coronary arteries presents diagnostic and therapeutic challenges. Myocardial infarction with non-obstructive coronary arteries (MINOCA) is a relatively uncommon but clinically significant entity, often caused by coronary vasospasm, microvascular dysfunction, or plaque erosion, requiring careful differentiation from other ischemic and non-ischemic causes.

We report the case of a 27-year-old Moroccan man with a high cardiovascular risk profile, including newly diagnosed type 2 diabetes, obesity, active smoking, and a family history of coronary artery disease, who presented to the emergency department with severe, persistent chest pain and ST-segment elevation on electrocardiography (ECG). Given the ST-segment elevation MI (STEMI)-mimicking presentation and lack of immediate angiography access, fibrinolysis was administered, though symptom persistence later suggested MINOCA. An angiography was conducted, revealing no obstructive coronary lesions. Further investigation with cardiac magnetic resonance imaging (MRI) confirmed an apical transmural MI. The patient's course was managed with dual antiplatelet therapy, beta-blockers, angiotensin-converting enzyme (ACE) inhibitors, and statins, with anti-anginal agents added upon the recurrence of angina.

This case illustrates the diagnostic complexities of MINOCA in young adults with ACS presentations and highlights the importance of advanced imaging to confirm MI and guide treatment. Future research should address optimal acute management (e.g., fibrinolysis vs. conservative strategies) and the role of intracoronary imaging (intravascular ultrasound (IVUS)/optical coherence tomography (OCT)) in MINOCA.

## Introduction

Acute coronary syndrome (ACS) in young adults presents unique diagnostic challenges, particularly when angiography reveals non-obstructive coronary arteries (<50% stenosis according to current guidelines) [1]. Myocardial infarction with non-obstructive coronary arteries (MINOCA) accounts for 5%-15% of acute MI (AMI) cases, with a predilection for women and younger populations [2]. Unlike atherosclerotic MI, MINOCA encompasses diverse mechanisms, including plaque disruption without obstruction, coronary vasospasm, microvascular dysfunction, and spontaneous coronary artery dissection (particularly in women <50) [1,2]. This heterogeneity demands a paradigm shift from conventional ACS protocols, as emphasized by Gulati et al. [1]: "MINOCA requires dedicated diagnostic pathways integrating advanced imaging and provocative testing where available." Wieczorkiewicz et al. [2] further demonstrate that up to 30% of MINOCA cases are initially misclassified, underscoring the need for heightened clinical suspicion in young patients with ischemic symptoms but clean coronaries.

We present an illustrative case of a 27-year-old man with multiple traditional risk factors (type 2 diabetes, obesity, smoking) whose STEMI-mimicking presentation revealed three critical gaps in MINOCA care: (1) the futility of fibrinolysis in non-thrombotic subtypes, (2) the diagnostic imperative of early cardiac MRI when angiographic-ECG discordance exists, and (3) the underrecognized burden of microvascular dysfunction in young males, a population less studied in MINOCA cohorts [1,2]. This case highlights the challenges of managing MINOCA in resource-limited settings where advanced imaging remains scarce.

## Case presentation

We present the case of a 27-year-old Moroccan man with significant cardiovascular risk factors, including recently diagnosed type 2 diabetes (hemoglobin A1c (HbA1c) 13.8%), obesity (body mass index (BMI) 33.5), smoking, and a family history of coronary artery disease.

The patient presented to the emergency department (ED) with epigastric pain, rated 7/10 on the Visual Analogue Scale (VAS), which began suddenly at rest, unrelated to exertion or food intake. Physical examination was unremarkable aside from tachycardia, and the initial ECG appeared normal. Troponin levels were mildly elevated (0.78 ng/mL) but normalized (0.04 ng/mL) following spontaneous pain resolution.

**Figure 1:**
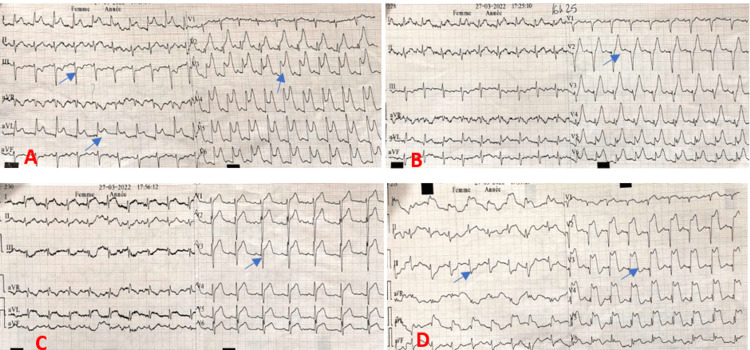
Time-evolution of ECG changes: (A) Baseline (pain onset) showing anterior ST-elevation (arrows) and reciprocal inferior ST-depression; (B) 10 minutes after presentation showing ample T waves; (C) 15 minutes after presentation demonstrating anterior ST-elevation (arrows) and reciprocal inferior ST-depression; (D) one-hour tracing showing Q-wave development with persistent ST-elevation.

However, six hours later (while still at the ED), he experienced more intense retrosternal constrictive (non-pleuritic) chest pain rated 10/10, persistent for more than 30 minutes, accompanied by cold sweats, nausea, and vomiting. ECG within 10 minutes (Figure 1) showed anterior ST-segment elevation with reciprocal inferior changes, evolving to large T waves and infarct Q waves, indicating a high likelihood of ACS.

The patient was admitted to the intensive care unit (ICU) with stable vital signs but ongoing severe pain. A bedside echocardiography demonstrated wall motion abnormalities and left ventricular (LV) dysfunction (LVEDD 45 mm, LVESD 35 mm, E/e' ratio 10).

The main diagnoses included STEMI, coronary spasm, and myocarditis.

Analgesics, including morphine, provided some pain relief. A nitroglycerin patch was applied to target possible coronary spasm, but there was no improvement, with continued chest pain and persistent ST-segment elevation on ECG. Due to the unavailability of a catheterization lab within 120 minutes of first medical contact (FMC), fibrinolysis was initiated, unfortunately without successful symptom resolution.

Laboratory test results (obtained two hours later) showed elevated troponin (0.04 ng/mL at 0 h → peak 5 ng/mL at 6 h → 1.8 ng/mL at 24 h) and leukocytosis, while C-reactive protein (CRP) and hemoglobin levels were within normal limits.

**Figure 2:**
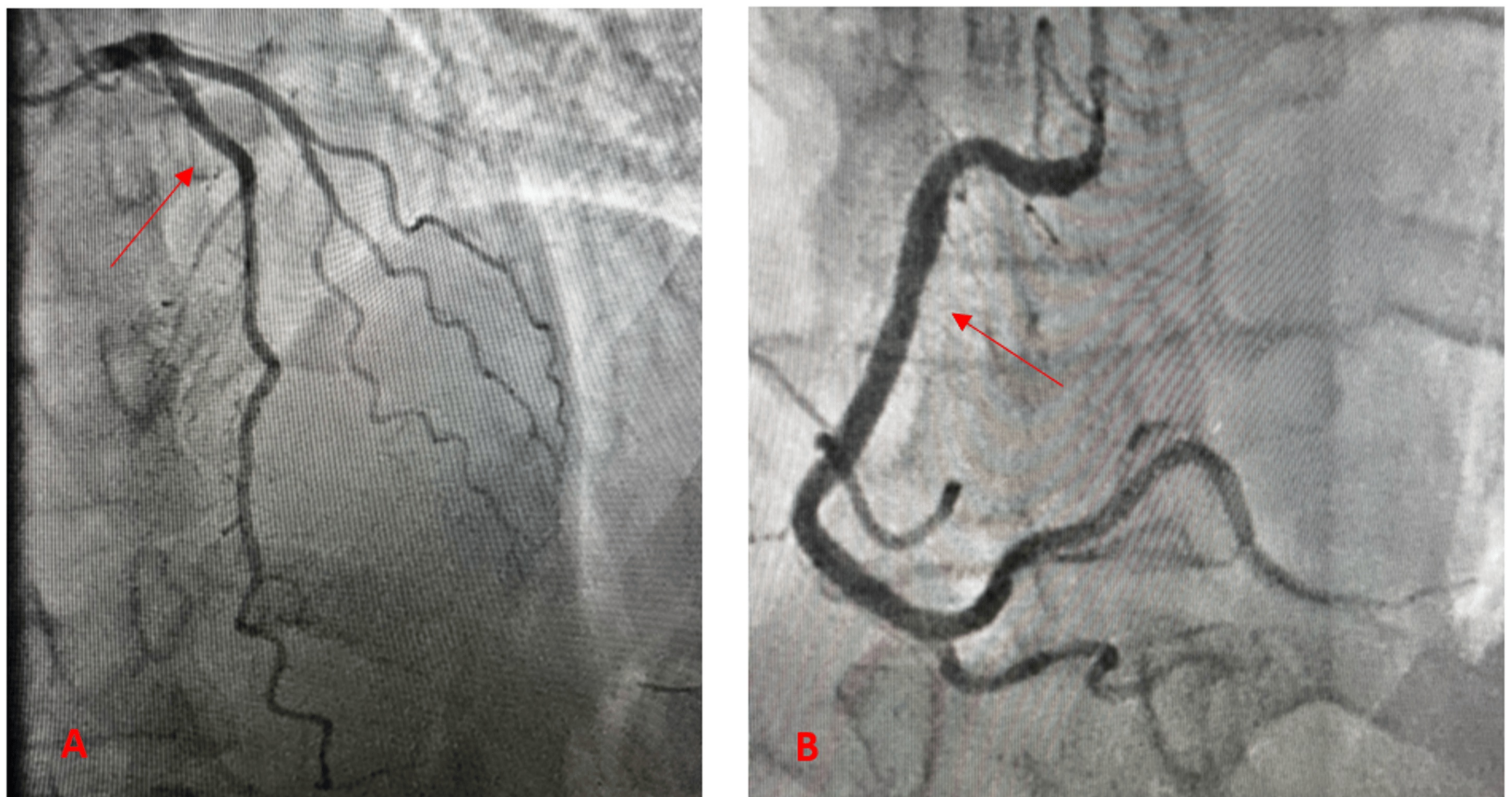
Coronary angiography images showing the cranial view of the normal left anterior descending artery (LAD) (A) and the left anterior oblique (LAO) view of the normal right coronary artery (RCA) (B). Normal angiography cannot exclude microvascular dysfunction or vasospasm.

Coronary angiography 24 hours after the initial presentation (Figure 2) revealed normal coronary arteries, ruling out obstructive coronary disease and coronary spasm. A diagnosis of myocarditis was also considered based on clinical findings.

Surprisingly, subsequent cardiac MRI revealed transmural late gadolinium enhancement (LGE) in the apical segments, confirming infarction. T2-weighted imaging showed focal apical edema, while the absence of mid-wall/epicardial LGE excluded myocarditis. Associated apical hypokinesis and preserved pericardial integrity further supported ischemic MINOCA, indicating a significant discrepancy with the angiography results.

Extended workup for secondary MINOCA causes was unremarkable, including negative antiphospholipid antibodies and normal protein C/S activity (ruling out thrombophilia), negative autoimmune markers (antinuclear antibody (ANA), anti-double-stranded DNA (anti-dsDNA), antineutrophil cytoplasmic antibody (ANCA)) with normal CRP, and a negative urine screen for cocaine/amphetamines.

Table 1 provides the key features of MINOCA.

**Table 1:** Differential diagnosis of MINOCA: key features. LGE: late gadolinium enhancement.

Feature	Ischemic (our case)	Non-ischemic (myocarditis)
LGE pattern	Subendocardial-transmural	Mid-wall/epicardial
Troponin kinetics	Rapid rise/fall	Prolonged elevation
ECG changes	Territorial (V1-V4)	Diffuse/dynamic

**Figure 3:**
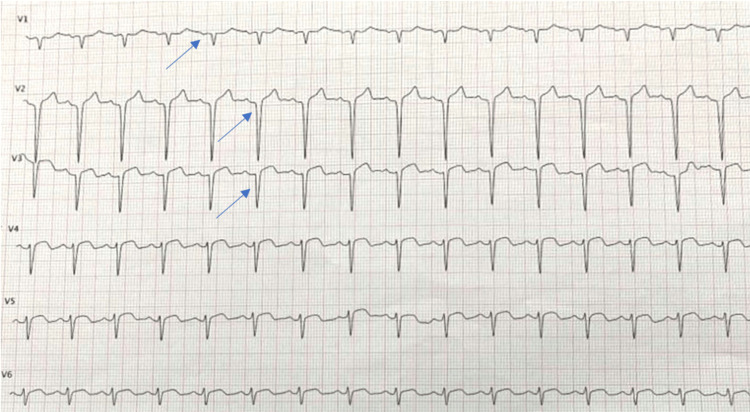
Post-discharge ECG showing persistent ischemic sequelae. The arrows indicate pathological Q waves in leads V1, V2, and V3. Persistent Q-waves in V1-V3 correlate with apical hypokinesis on follow-up echo (EF 40%).

Following discharge, our patient initially experienced symptomatic improvement but developed recurrent angina (Canadian Cardiovascular Society Class III) two weeks later. Subsequent ECG (Figure 3) showed no new ischemic changes, though echocardiography indicated persistent wall motion abnormalities and LV dysfunction (ejection fraction (EF) 40%). Trimetazidine and diltiazem were added to the patient’s treatment with slight improvement of symptoms.

## Discussion

This case highlights the challenges of diagnosing and managing ACS in young patients with significant risk factors but non-obstructive coronary arteries. Studies by Gulati et al. and Wieczorkiewicz et al. emphasize differentiating ischemic causes (e.g., coronary vasospasm, spontaneous coronary artery dissection (SCAD)) from non-ischemic ones (e.g., myocarditis) in ST-elevation cases [1,2]. This case adds to the spectrum of atypical MINOCA presentations, as recently synthesized by Khan et al., who emphasized diagnostic vigilance in young patients with misleading symptoms and clean angiograms [3]. 

The VIRGO study reports MINOCA in ~11% of young AMI patients, predominantly women, often involving non-plaque mechanisms like vasospasm, SCAD, or embolization. Despite fewer traditional risk factors, MINOCA outcomes at 12 months are comparable to MI-CAD [4-7].

In our case, the coronary angiography revealed normal coronary arteries, a key finding that excluded obstructive coronary artery disease as the cause of the AMI. However, this result also raised a diagnostic challenge by necessitating further investigations into potential non-obstructive mechanisms. While conventional angiography is invaluable in evaluating acute coronary syndromes, it has limitations in detecting conditions such as coronary vasospasm, microvascular dysfunction, or SCAD.

Advanced imaging (IVUS/OCT) could have identified subtle abnormalities, such as intimal tears, intramural hematoma, or plaque disruption, which may have been missed with angiography alone. However, these tools, along with vasospasm provocation testing, remain underutilized despite their diagnostic value [8-10].

In this case, CMR played a pivotal role by revealing ischemic injury undetectable on angiography, reinforcing its utility in differentiating ischemic from inflammatory processes, as highlighted by Dastidar et al. [11,12].

While SCAD was considered given the patient's age, its likelihood was low due to the absence of angiographic hallmarks (luminal irregularities, intimal flaps), no predisposing factors (fibromuscular dysplasia, postpartum status), and MRI showing transmural LGE following coronary territories rather than SCAD's typical branch-specific patterns.

Microvascular dysfunction emerged as the probable mechanism in our case, supported by recurrent angina despite patent epicardial arteries, MRI demonstrating subendocardial-to-transmural LGE (microvascular injury pattern), and exclusion of other etiologies. This aligns with current pathophysiological models of MINOCA that highlight the heterogeneity of mechanisms and support tailored diagnostic strategies [13,14]. While non-invasive CFR assessment (stress echocardiography) was planned, logistical delays occurred.

The management approach in this case balanced ACS treatment urgency with diagnostic uncertainties. A nitroglycerin trial was initiated for potential vasospasm, but persistent ST-elevation necessitated urgent reperfusion per guidelines. Fibrinolysis was administered based on STEMI-equivalent ECG, ongoing ischemic pain, and lack of immediate percutaneous coronary intervention (PCI) (<120 min FMC-to-balloon) but proved of limited efficacy.

In retrospect, earlier angiography with adjunctive imaging could have avoided thrombolysis, identified non-atherosclerotic mechanisms, and guided targeted therapy. This experience highlights the critical need for protocolized approaches to ambiguous STEMI cases in non-PCI centers, and health system investments to improve intracoronary imaging access for MINOCA diagnosis.

While ACS protocols guide acute management, long-term strategies must be individualized. Beta-blockers and ACE inhibitors were selectively prescribed based on verified indications (LV dysfunction (EF 40%) and recurrent angina) rather than empirically applied. Juan-Salvadores et al. suggest these medications may offer prognostic benefits in MINOCA, though data in young cohorts remains limited [13]. Young MINOCA patients face elevated MACE risk, necessitating aggressive secondary prevention.

Long-term prognosis in young MINOCA patients is variable, with a risk of recurrent ischemic events and lingering LV dysfunction [6,13,15]. Given the patient's recurrent angina with persistent functional limitations (6 MWD 380 m), a common complication with substantial quality-of-life impacts, management focused on available modalities: stress cardiac MRI (planned to quantify microvascular ischemia) and pharmacologic optimization (ranolazine or intensified calcium channel blockade if symptoms persist).

This pragmatic approach aligns with the VIRGO study that emphasized the importance of a detailed follow-up, including comprehensive imaging with CMR, to assess myocardial viability and detect subclinical scarring [6]. Similarly, Juan-Salvadores et al. highlighted the variability in pharmacologic management and the gap in standardized care for young MINOCA patients, stressing the need for prospective trials to guide therapy [13].

Psychosocial factors also play a role. Depression and stress impact recovery and recurrence, necessitating a holistic approach [9]. This case underscores the importance of long-term monitoring, symptom management, and tailored therapy in young MINOCA patients.

Limitations

Our findings are constrained by unavailable IVUS/OCT, which may have detected occult plaque pathology. The absence of psychological support may have impacted emotional recovery, given MINOCA’s association with PTSD/depression [9]. Follow-up challenges in young patients were evident, with incomplete three-month functional data due to missed visits. These limitations highlight the need for improved resource allocation in MINOCA care.

Research priorities

Key research directions include evaluating fibrinolysis versus conservative management in STEMI-mimicking MINOCA, integrating IVUS/OCT for angiography-negative MI, and exploring anti-inflammatory therapies for microvascular dysfunction.

## Conclusions

This case emphasizes three clinical lessons: first, when ST-elevation and angiographic findings are discordant, early cardiac MRI is essential; second, the limited effectiveness of fibrinolysis in MINOCA highlights the need for tailored acute strategies; and third, attention should be given to microvascular dysfunction and psychosocial stressors in young patients presenting with MINOCA.
